# C:N:P Stoichiometry and Leaf Traits of Halophytes in an Arid Saline Environment, Northwest China

**DOI:** 10.1371/journal.pone.0119935

**Published:** 2015-03-23

**Authors:** Lilong Wang, Guanxiang Zhao, Meng Li, Mingting Zhang, Lifang Zhang, Xinfang Zhang, Lizhe An, Shijian Xu

**Affiliations:** MOE Key Laboratory of Cell Activities and Stress Adaptations, School of Life Sciences, Lanzhou University, Lanzhou, China; University of Copenhagen, DENMARK

## Abstract

Salinization is an important and increasingly prevalent issue which has broad and profound effects on plant survival and distribution pattern. To understand the patterns and potential drivers of leaf traits in saline environments, we determined the soil properties, leaf morphological traits (specific leaf area, SLA, and leaf dry matter content, LDMC), leaf chemical traits (leaf carbon, C, nitrogen, N, and phosphorus, P, stoichiometry) based on 142 observations collected from 23 sites in an arid saline environment, which is a vulnerable ecosystem in northwest China. We also explored the relationships among leaf traits, the responses of leaf traits, and plant functional groups (herb, woody, and succulent woody) to various saline environments. The arid desert halophytes were characterized by lower leaf C and SLA levels, higher N, but stable P and N:P. The leaf morphological traits were correlated significantly with the C, N, and P contents across all observations, but they differed within each functional group. Succulent woody plants had the lowest leaf C and highest leaf N levels among the three functional groups. The growth of halophytes might be more limited by N rather than P in the study area. GLM analysis demonstrated that the soil available nutrients and plant functional groups, but not salinity, were potential drivers of leaf C:N:P stoichiometry in halophytes, whereas species differences accounted for the largest contributions to leaf morphological variations. Our study provides baseline information to facilitate the management and restoration of arid saline desert ecosystem.

## Introduction

Salinization is a major environmental and agricultural problem throughout the world, which reduces soil productivity and leads to desertification, especially in arid and semiarid regions [1]. According to a report published by the FAO in 2000, the total global area affected by salinity was 831 million hectares, which encompassed over 100 countries in Africa, Asia, Australasia, and the Americas [2]. The excess accumulation of salt in soil imposes physiological constraints on plants, including osmotic stress, ionic imbalance, oxidative stress, and disturbance of photosynthesis, thereby affects plant growth [3–5]. This situation has been exacerbated because of the effects of land over-exploitation by humans, and the original scarcity of water at desert—oasis ecotones in arid and semiarid regions [6, 7]. Therefore, it is important to understand the physiological and structural mechanisms [8, 9], nutrient uptake and distribution patterns [10, 11] of desert halophytes in saline environments.

The specific leaf area (SLA, i.e., the ratio of the leaf area relative to the leaf dry mass) and leaf dry matter content (LDMC, i.e., the ratio of the leaf dry mass relative to the saturated fresh mass) [12] have been analyzed as key leaf traits in numerous studies [13, 14] because they can provide general information about plant growth and the broad spectrum of leaf investment strategies. The SLA reflects the capacity for resource acquisition and it has been shown to be strongly correlated with the relative growth rate, net photosynthetic rate, and leaf life span [15, 16]. Studies have shown that species with a low SLA are more adapted to resource-poor and arid environments [12]. The LDMC has been shown to be the best single variable for locating non-succulent species on a resource use axis, whereas the SLA is more suitable for succulents [12, 17].

Chemical elements are the fundamental components of molecules, cells, individuals, communities, and even the biosphere, and they are the basic principles of ecology and stoichiometry [18]. Ecological stoichiometry is an integrative approach, which yields new insights into the trophic dynamics, biogeochemical cycling, and biodiversity within or among individual species, populations, communities, and ecosystems [19–21]. Compared with phytoplankton, terrestrial plants grow in a wider range of nutrient conditions, and they exhibit a higher degree of “stoichiometric homeostasis” in terms of the major nutrients: C, N, and P [20]. The C:N:P stoichiometry of terrestrial plants can reflect how well a plant is adjusted to the local growth conditions [22], thus many studies have focused on the stoichiometry of elements in plants in relation to broad-scale variations in geographical and climatic factors [23–25]. However, to the best of our knowledge, few studies have focused on the effects of salinity on the C:N:P stoichiometry of halophytes, especially the relationship between leaf morphological traits and nutrient stoichiometry in field environments in arid saline regions. Thus in this study, we determined the leaf traits, leaf C:N:P stoichiometry of halophytes, as well as the soil properties along natural salinity gradients in an arid environment in northwest China. In particular, the following issues were addressed: (1) the patterns of leaf traits, C:N:P stoichiometry, and their relationships in desert halophytes; (2) the response of the leaf traits and C:N:P stoichiometry of halophytes to various salinity levels; and (3) the roles of soil properties and plant functional groups in structuring the leaf C, N, and P contents of desert halophytes in the study area.

## Materials and Methods

### Ethics statement

This study was authorized and assisted by the Water Resources Administration of Shule River and the Anxi Extremely-Arid Desert National Nature Reserve. We confirmed that the field studies did not involve endangered or protected species, and declare that the work reported here complies with the current laws of China.

### Site description

The present study was conducted in an arid environment in northwest China (39°57′26"–40°27′19"N, 96°48′46"–97° 3′54"E), which has a mean annual temperature of 6.8–9°C, mean annual precipitation of 46–62 mm, and a mean annual evaporation capacity of 1500–2500 mm, with a typical temperate continental climate. The high evaporation-precipitation ratio in this area has generated severe salinity [26], thereby leading to low species diversity, which mainly comprises desert halophytes. Given the uniform mean annual precipitation and temperature in the study area, salinity is the major factor that affects the distribution and growth of plants. Thus, this area provides a unique opportunity to explore the patterns and drivers of the leaf traits and C:N:P stoichiometry of desert halophytes in a saline field environment.

### Sampling and methods

The study area has been protected by the Water Resources Administration of Shule River and the Anxi Extremely-Arid Desert National Nature Reserve for nearly 20 years. We selected sites with relatively stable salinity in previous years based on our own investigation and published data [26, 27]. Finally, 23 sites were selected with different levels of salinization, as well as minimal grazing and anthropogenic disturbances (S1 Table). Samples were collected and measurements were performed during the growing season in July 2013. Based on a 20 × 20 m plot, topsoil samples (0–20 cm) and the leaves were selected for measurement at each site. In total, 142 observations of 18 desert halophyte species were obtained in our dataset (S2 Table). The plant samples were divided into three functional groups according to the descriptions in *Halophytes in China* [28], i.e., herb (herb species with non-succulent leaves), woody (woody species with non-succulent leaves), and succulent woody (woody species with succulent leaves), and all of the species were deciduous and perennial.

Sun-exposed and fully expanded mature leaves (or leafy shoots) were collected from five individuals of each species (total fresh mass >100 g for each species) at each site, which were mixed uniformly and placed in paper envelopes (a bag for one species) for subsequent analyses. The fresh leaves were rehydrated in the dark for 12 h, oven dried at 60°C, and the dry weight was divided by the saturated weight to calculate the LDMC as g g^–1^ [29]. The SLA was measured using the photographic method. Fresh leaves were placed on a white background with a ruler and photographed using a camera (D5100, Nikon Corporation, Japan) mounted on a tripod, with illumination from two lamps on different sides [29]. After taking the photographs, the leaves were oven dried at 60°C and the photographs were analyzed using Image J 1.45s (National Institutes of Health, USA, http://imagej.nih.gov/ij). The SLA (as m2 kg^–1^) was obtained by calculating the leaf area:dry weight ratio. Triplicate soil samples (0–20 cm) were obtained using a soil auger at each site, where each replicate (not less than 200 g) comprised a mixture of at least three cores. Fresh soil was placed in an aluminum box and weighed in situ using an electronic balance, and then dried at 105°C for 24 h in the laboratory to determine the soil water contents. Next, the air-dried soil samples were passed through a 0.25 mm sieve to remove gravel and plant remnants, before grinding into a fine powder using a ball mill (MM200, Retsch, Haan, Germany), where the ball mill was cleaned completely after each sample. The total C and N concentrations in the leaves and soils were measured using an elemental analyzer (FLASHEA 1112 Series CNS Analyzer, Thermo, USA). The total P concentrations were measured using the ammonium molybdate method after persulfate oxidation [30]. The mass-based leaf N and P contents were expressed as N or leaf N, and P or leaf P. N_area_ and P_area_ denote the area-based N and P contents, respectively. Soil extractable nitrogen (SEN) was determined photometrically at one site KCl (2 mol L^-1^) extractions using a flow analysis system (FIAstar 5000, FOSS, DK). To determine the soil extractable phosphorus (SEP), soil samples were extracted with 0.5 mol L^-1^ NaHCO_3_, filtered, and analyzed to detect orthophosphate by reacting with acid molybdate and reducting with ascorbic acid [31]. The soil pH was measured using a soil:water ratio of 1:2.5 (weigh/weight) with a pH electrode (IQ 150, Spectrum, USA).

The soil electrical conductivity (EC) can be used to obtain accurate estimates of the soil salinity [32]. In this study, we used a soil:water ratio of 1:5 (weight/weight), which is known to be a simple and time-saving method for analyzing saline soils [33]. Thus, soil (air dried, 10 g, 2-mm sieved) and 50 mL deionized water were mixed on a mechanical shaker for 15 min (180 rpm), allowed to settle for at least 1 h, agitated again for 5 min, filtered through a 0.45 μm filter paper, and measured with an EC meter (2220, Spectrum, USA).

### Data analysis

All of the variables were log-transformed to normalize the distribution of the data. The transformed data were analyzed at two levels: (1) all of the data (treating all observations together); and (2) averaging by species within each functional group (treating the three functional groups of herb, woody and succulent woody separately). One-way ANOVA was used to explore the differences in the SLA, LDMC, and C:N:P stoichiometry between functional groups. An independent-samples *t*-test was employed to compare the C:N:P stoichiometry patterns with previous studies. We analyzed the relationships between leaf traits and C:N:P stoichiometry, and the trends in the leaf traits and C:N:P stoichiometry along the salinity gradients using ordinary least squares regression (OLS, model I regression). Based on a cluster analysis, 23 sites were classified according to four salinity levels. General linear models (GLMs) was used to quantify the effects of soil nutrients, soil salinity, and plant functional groups on leaf C, N, and P contents, SLA, and LDMC. The statistical analyses were performed using SPSS 18.0 (SPSS Inc. USA) and figures were obtained with Origin 8.0 (OriginLab Corporation, USA).

## Results

### Leaf C:N:P stoichiometry patterns and leaf traits

**Fig 1:**
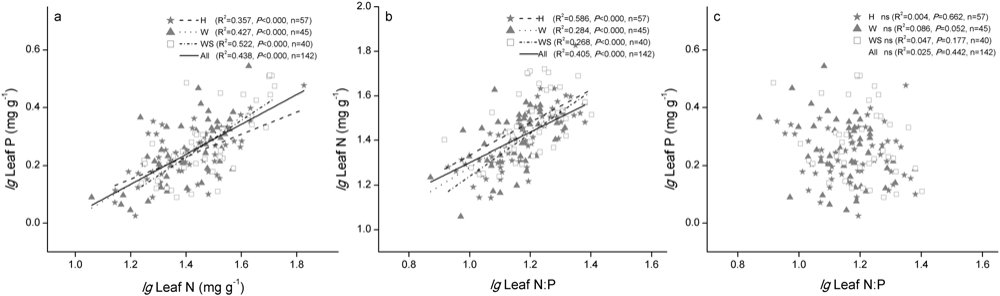
Relationship between leaf N, P and N:P. H: herb species, W: woody species with non-succulent leaves, WS: woody species with succulent leaves. Lines are shown if the linear regressions were significant at *P* < 0.05, n: number of samples, ns: no significant correlation. The N and P content are mass-based (mg g^–1^).

For all species, the mean leaf C, N, and P contents, C:N, C:P, and N:P ratios were 369.72 mg g^–1^, 28.12 mg g^–1^, 1.85 mg g^–1^, 15.69, 229.44, and 15.37 respectively, and the mean SLA and LDMC were 8.60 m2 kg^–1^ and 0.28 g g^–1^ respectively (Table 1). The mean leaf C of the halophytes was significantly lower than that of the grassland biomes of China and of global terrestrial plants [20, 34] (Table 2). The mean leaf P was similar to that of the grassland biomes of China and global averages, as well as that in typical desert and desertified regions of north China [35], but significantly higher than the Chinese flora [23] (Table 2). The mean leaf N of desert halophytes was significantly higher than that of the desert steppe plants of China’s grasslands (25.7 mg g^-1^) [36], global means [25], and other datasets (Table 2). Across all observations and within each functional group, the leaf N and P were highly positively correlated (R^2^ = 0.433, *P* < 0.0001), and the leaf N:P was highly positively correlated with leaf N, but not with leaf P (Fig. 1). The lowest leaf C content and highest N content were found in the succulent woody group (Table 1), but there were no significant differences in C, N, C:N, and C:P between the herb and woody groups. Leaf P and N:P were significantly higher in the succulent woody plants than herbs (*P* < 0.05), and the N:P ratios of the herb and woody groups were stable (*P* > 0.05) (Table 1). The mean SLA value of woody plants (7.79 m2 kg^–1^) was similar (*P* > 0.05) to that of succulent woody plants (6.94 m2 kg^–1^), but both were lower than that of herbs (10.41 m2 kg^–1^, *P* < 0.05). The LDMC values of herb (0.33 g g^–1^) and woody plants (0.31g g^–1^) were similar (*P* > 0.05), but they were significantly higher than those of succulent woody plant (0.17 g g^–1^, *P* < 0.05, Table 1).

**Table 1:** Leaf traits and leaf C, N, P stoichiometry for all observation and within functional groups.

	Overall			Herb			Woody			Succulent woody		
	Mean	SD	CV	Mean	SD	CV	Mean	SD	CV	Mean	SD	CV
C	396.72	45.37	0.11	421.22a	22.0	0.05	411.22a	33.2	0.08	345.49b	41.2	0.12
N	28.12	9.42	0.34	26.76b	9.30	0.35	26.39b	7.16	0.27	31.99a	10.8	0.34
P	1.85	0.50	0.27	1.74b	0.37	0.21	1.85ab	0.49	0.26	1.99a	0.62	0.31
C:N	15.69	5.62	0.36	17.33a	5.31	0.31	17.01a	5.92	0.35	11.87b	3.68	0.31
C:P	229.44	63.70	0.28	252.17a	54.9	0.22	238.10a	65.2	0.27	187.29b	53.7	0.29
N:P	15.37	3.69	0.24	15.37a	3.88	0.25	14.56a	3.28	0.23	16.29a	3.74	0.23
SLA	8.60	3.00	0.34	10.41a	3.05	0.29	7.79b	1.95	0.25	6.94b	2.36	0.34
LDMC	0.28	0.09	0.34	0.33a	0.06	0.18	0.31a	0.08	0.26	0.17b	0.07	0.41

Mean value (Mean), standard deviation (SD), coefficient of variation (CV, defined as SD/Mean). SLA (m2 kg^-1^), LDMC (g g^–1^), and leaf C, N, P (mg g^–1^), C:N, C:P, N:P (on dry mass basis). Differences between groups were tested using a one-way ANOVA with Turkey’s post hoc test. Significance at *P* < 0.05 are indicated by different letters.

**Table 2:** Comparison between the Leaf C, N, and P and C:N, C:P, and N:P in this study and other datasets.

Data source	C(mg g^–1^)	N(mg g^–1^)	P(mg g^–1^)	C:N(mass)	C:P(mass)	N:P(mass)
This study						
Mean±SD	396.7±45.4	28.1±9.4	1.85±0.5	15.7±5.6	229.4±63.7	15.4±3.7
n	142	142	142	142	142	142
Yu-lin (2010)						
Mean±SD	—	24.5±8.1*	1.74±0.9	—	—	15.8±7.5
n	—	214	214	—	—	214
Jing-sheng (2006, 2008)						
Mean±SD	442.4±28.1*	26.5±8.5*	1.91±0.84	18.4±5.8*	275.2±116.1*	15.3±5.2
n	526	526	526	526	526	526
Wen-Xuan (2005)						
Mean±SD	—	20.2±8.4*	1.46±1.0*	—	—	16.3±9.3*
n	—	554	745	—	—	547
Reich (2004) and Elser (2000)						
Mean±SD	461.6±72.2*	20.1±8.7*	1.77±1.1	23.8±17.3*	300.9±236.8*	13.8±9.5*
n	76	1251	932	62	43	894

* Significance between this study and another data source (*P*<0.001)

### Relationships among SLA, LDMC, and leaf nutrients

**Fig 2:**
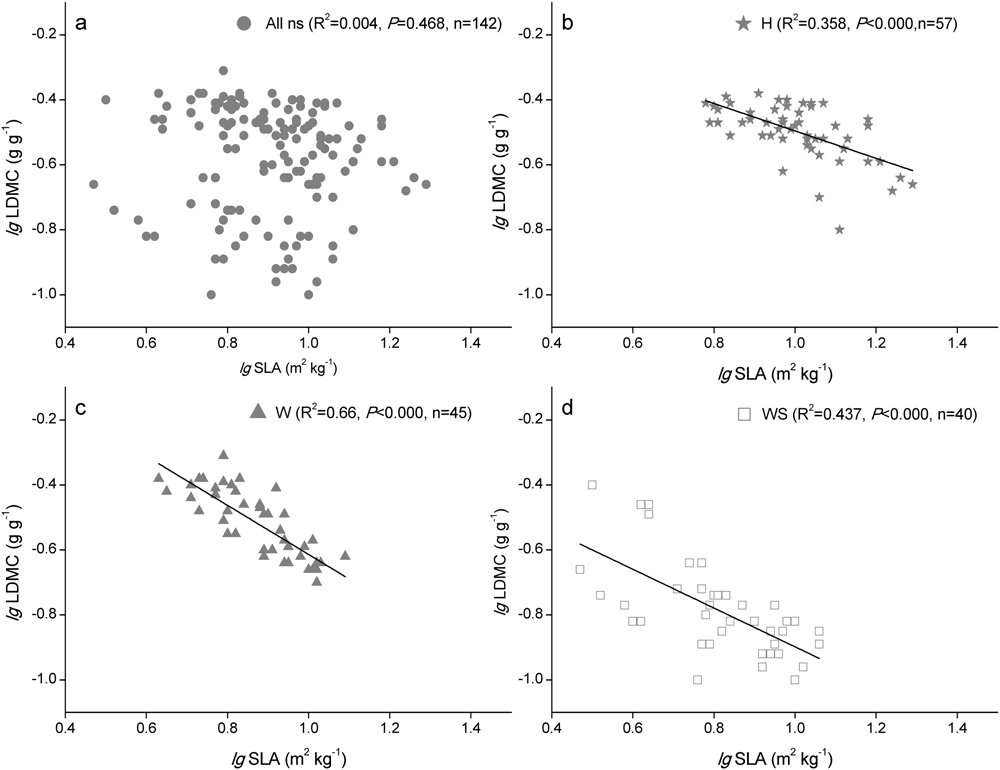
Relationships between the SLA and the LDMC in three functional groups. H: herb species, W: woody species with non-succulent leaves, WS: woody species with succulent leaves. Lines are shown if the linear regressions were significant at *P* < 0.05, n: number of samples, ns: no significant correlation.

**Fig 3:**
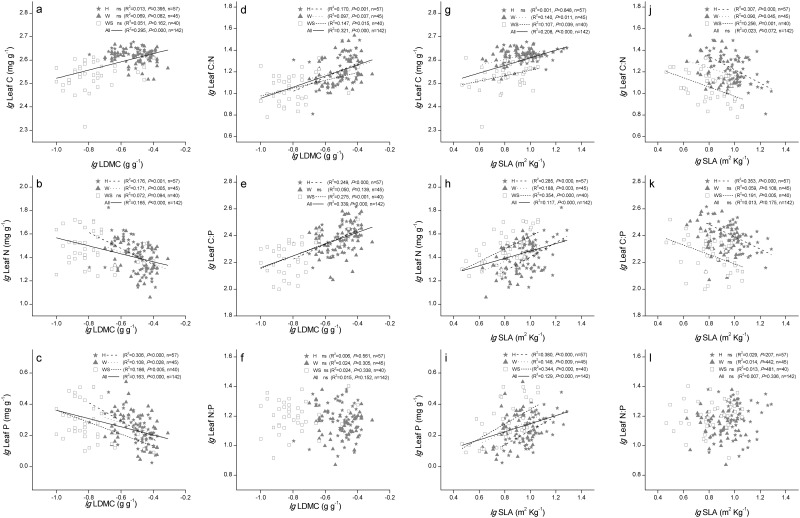
Relationships between the leaf morphological traits and leaf nutrient stoichiometry. H: herb species, W: woody species with non-succulent leaves, WS: woody species with succulent leaves. Lines are shown if the linear regressions were significant at *P* < 0.05, n: number of samples, ns: no significant correlation. The C, N, and P contents are mass-based (mg g^–1^).

**Fig 4:**
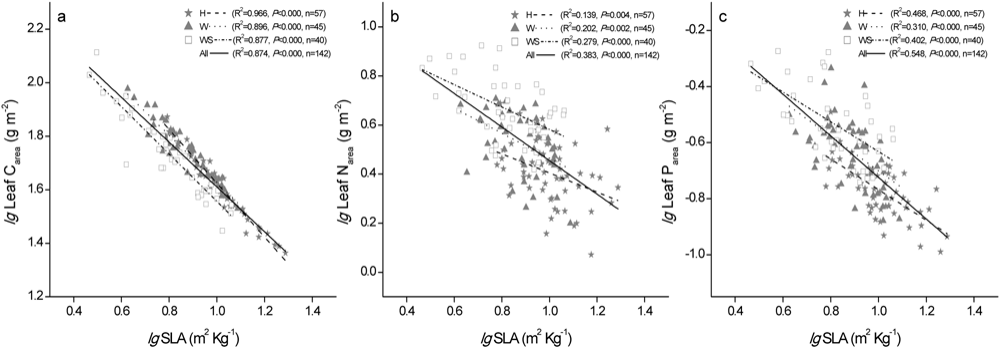
Relationships between SLA and leaf area-based leaf C, N, and P contents. H: herb species, W: woody species with non-succulent leaves, WS: woody species with succulent leaves. Lines are shown if the linear regressions were significant at *P* < 0.05, n: number of samples. The C, N, P contents are area-based (m^2^ kg^–1^).

SLA and LDMC were not correlated across all observations (R^2^ = 0.004, *P* = 0.468, Fig. 2a), whereas they were highly negatively correlated within each of the three functional groups (*P* < 0.0001, Fig. 2). Leaf C was correlated positively with LDMC and SLA across all observations (*P* < 0.0001, Fig. 3a, 3g), but there were no significant correlations between leaf C and LDMC within each group (*P* > 0.05, Fig. 3a). There were significant correlations between leaf N and leaf morphological traits (Fig. 3b, 3h), except in the succulent woody group (Fig. 3b). Across all observations and within each group, LDMC and SLA were significantly correlated with leaf P (Fig. 3c, 3i), but they were not correlated with leaf N:P (Fig. 3f, 3l). There were more significant negative correlations between SLA and leaf C_(area)_, N_(area)_, and P_(area)_ compared with those between SLA and the mass-based leaf C, N, and P (Fig. 4).

### Variations in the leaf C:N:P stoichiometry and leaf traits with the salinity gradients

There were no significant trends in many leaf variables within the three functional groups along the salinity gradients based on the regression analysis (Table 3). However, the leaf C decreased slightly in the woody group and the leaf N:P increased slightly in both the woody and succulent woody plants (Table 3). For individual species with widespread distributions in the study area, such as *Phragmites australis* (herb), *Tamarix leptostachya* (woody plant with non-succulent leaves) and *Lycium ruthenicum* (woody plant with succulent leaves), there were no significant trends in many of the leaf variables along the salinity gradients, although the leaf C and C:N decreased slightly in *Lycium ruthenicum* (Table 4).

**Table 3:** Summary of regression analyses for the three functional groups along salinity gradients.

Bivariate	H (n = 57)				W (n = 45)				WS (n = 40)			
	R^2^	Intercept	Slope	*P*	R^2^	Intercept	Slope	*P*	R^2^	Intercept	Slope	*P*
Salinity gradients *vs*. Leaf C	0.02	2.61	0.00	0.30	0.10	2.64	-0.01	0.03**	0.06	2.56	-0.01	0.12
Salinity gradients *vs*. Leaf N	0.00	1.39	0.01	0.65	0.02	1.36	0.02	0.31	0.05	1.42	0.03	0.16
Salinity gradients *vs*. Leaf P	0.03	0.20	0.02	0.20	0.05	0.31	-0.02	0.14	0.00	0.29	0.00	0.87
Salinity gradients *vs*. Leaf C:N	0.01	1.24	-0.01	0.52	0.05	1.27	-0.03	0.14	0.12	1.14	-0.04	0.03*
Salinity gradients *vs*. Leaf C:P	0.04	2.43	-0.02	0.14	0.01	2.33	0.01	0.47	0.01	2.27	-0.01	0.62
Salinity gradients *vs*. Leaf N:P	0.00	1.19	-0.01	0.65	0.20	1.06	0.04	0.01**	0.13	1.14	0.03	0.02*
Salinity gradients *vs*. SLA	0.03	0.95	0.02	0.19	0.00	0.87	0.00	0.94	0.04	0.76	0.03	0.21
Salinity gradients *vs*. LDMC	0.01	-0.48	-0.01	0.57	0.02	-0.55	0.01	0.36	0.05	-0.73	-0.03	0.15

H, W, and WS represent herb, woody species with non-succulent leaves, and woody species with succulent leaves, respectively. The leaf C, N, and P contents are based on the dry mass (mg g^–1^). Significance at *P* < 0.05 and *P* < 0.01 (*p*-value of the slope) are indicated by * and **, respectively.

**Table 4:** Summary of regression analyses for three widely distributed species along salinity gradients.

Bivariate	*Pa* (H; n = 16)				*Tl* (W; n = 20)				*Lr* (WS; n = 16)			
	R^2^	Intercept	Slope	*P*	R^2^	Intercept	Slope	*P*	R^2^	Intercept	Slope	*P*
Salinity gradients *vs*. Leaf C	0.03	2.61	0.00	0.54	0.05	2.61	-0.01	0.32	0.44	2.58	-0.02	0.01**
Salinity gradients *vs*. Leaf N	0.10	1.36	0.02	0.22	0.16	1.27	0.04	0.09	0.18	1.36	0.04	0.1
Salinity gradients *vs*. Leaf P	0.23	0.15	0.03	0.06	0.01	0.21	0.01	0.72	0.01	0.23	0.01	0.67
Salinity gradients *vs*. Leaf C:N	0.12	1.26	-0.03	0.19	0.17	1.34	-0.05	0.07	0.33	1.23	-0.05	0.02*
Salinity gradients *vs*. Leaf C:P	0.22	2.47	-0.03	0.07	0.02	2.40	-0.02	0.56	0.09	2.35	-0.02	0.26
Salinity gradients *vs*. Leaf N:P	0.00	1.21	0.00	0.86	0.21	1.06	0.03	0.04*	0.08	1.13	0.03	0.3
Salinity gradients *vs*. SLA	0.02	1.00	0.01	0.64	0.22	0.69	0.03	0.04*	0.15	0.81	0.03	0.13
Salinity gradients *vs*. LDMC	0.12	-0.41	-0.02	0.18	0.06	-0.47	0.01	0.29	0.24	-0.78	0.01	0.05

*Pa*, *Phragmites australis; Tl*, *Tamarix leptostachya; Lr*, *Lycium ruthenicum* are three widely distributed species in the study area. The leaf C, N, and P contents are based on the dry mass (mg g^–1^). Significance at *P* < 0.05 and *P* < 0.01 (*p*-value of the slope) are indicated by * and **, respectively.

### Roles of plant functional groups and soil variables in terms of the leaf C, N, and P contents, SLA, and LDMC

**Fig 5:**
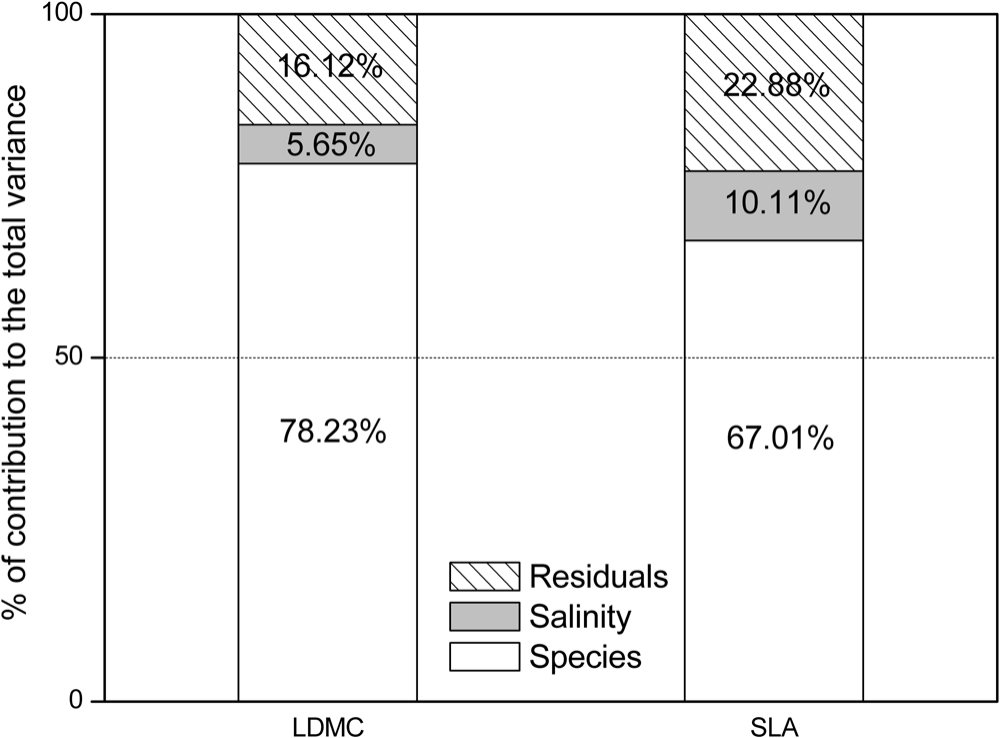
GLM analysis for the partitioning of the total variance of leaf morphological traits (SLA and LDMC) to the species and soil salinity.

Salinity made minor contributions to the variations in leaf nutrient traits (Table 5) and leaf morphological traits (Fig. 5). Soil total C accounted for 8.89% of the variation in leaf C, but plant functional groups explained 43.89% of this variation (Table 5). The soil extractable N, but not the total soil N was a significant factor that explained the variation in the leaf N (26.18% for soil extractable N and *P* > 0.05 for total soil N) (Table 5). The total soil P and soil extractable P were both significant factors that explained the variation in the leaf P. However, more of the variation in leaf P was explained by soil extractable P than the total soil P (Table 5). Species explained most of the variation in leaf SLA and LDMC (78.23% for LDMC and 67.01% for SLA).

**Table 5:** Effects of soil nutrient, functional groups, and salinity on the variations in leaf C, N, and P.

	Leaf C				Leaf N				Leaf P				Leaf N				Leaf P			
	d.f.	SS	*P*	SS%	d.f.	SS	*P*	SS%	d.f.	SS	*P*	SS%	d.f.	SS	*P*	SS%	d.f.	SS	*P*	SS%
Soil nutrient	STC				STN				STP				SEN				SEP			
	13	0.032	0.027*	8.89	12	0.168	0.472	12.81	19	0.703	<0.001**	38.21	19	0.698	0.004**	26.18	19	0.703	<0.001**	42.90
FG	2	0.158	<0.001**	43.89	2	0.059	0.134	4.50	2	0.025	0.199	1.36	2	0.064	0.141	2.40	2	0.025	0.199	1.52
Salinity	2	0.004	0.222	1.11	1	0.009	0.422	0.69	3	0.201	<0.001**	10.92	2	0.065	0.139	2.44	—	—	—	—
FG*Salinity	6	0.021	0.014**	5.83	6	0.053	0.711	4.04	6	0.068	0.187	3.70	6	0.049	0.806	1.84	6	0.068	0.187	4.15
Residuals	117	0.145		40.28	72	1.022		77.96	111	0.843		45.82	111	1.790		67.14	111	0.843		51.44

STC, soil total carbon; STN, soil total nitrogen; STP, soil total phosphorus; SEN, soil extractable nitrogen; SEP, soil extractable phosphorus; FG, functional groups; Salinity, soil EC_1:5_; d.f., degrees of freedom; SS, sum of squares; SS%, percentage of sum of squares explained.

*P < 0.05,

**P < 0.01.

## Discussion

In this study, we determined the leaf traits and C:N:P stoichiometry of desert halophytes in an arid saline habitat, which is a vulnerable ecosystem in northwest China. We demonstrated that the desert halophytes were characterized by low leaf C and SLA levels, high N, but stable P and N:P. The leaf morphological traits were correlated significantly with the C, N, and P contents across all observations, but there were different correlations within each functional group. Succulent woody plants had the lowest leaf C and highest leaf N levels compared with herb and woody plants. The GLM analysis showed that the leaf morphological trait and leaf C:N:P stoichiometry in the arid saline region were influenced by the plant functional group, soil available nutrients, while soil salinity had minor effects. The above results suggest that functional group and soil available nutrient levels, rather than individual species, should be considered more in the vegetation restoration and land management in saline systems.

### C:N:P stoichiometry and leaf trait patterns of halophytes

The low leaf C levels detected in this study may be attributable to the decreased plant productivity caused by salinization [37]. Salt stress usually inhibits photosynthesis by reducing stomatal conductance [38] and the water potential [4, 39], thereby decreasing C fixation. Moreover, plants need to consume additional energy to cope with salt stress [40, 41], which leads to increased metabolic costs [42]. Therefore, the lower C concentrations in halophyte leaves may be a trend associated with arid saline conditions.

The increased amount of N input caused by human activity is a major issue related to ecological sustainability that affects the N:P balance in ecological systems, especially in fragile ecosystems such as deserts [43, 44]. In natural conditions, the leaf N concentration has a positive linear relationship with soil N [45]. Even in a nutrient-rich environment, i.e., a field experiment where N was added to a semi-arid grassland, Lü et al. found increased levels of soil inorganic N resulted in a major increase in leaf N [46]. However, in the present study, irrespective of the low soil N content (S3 Table), higher leaf N levels were detected in halophytes, which is not consistent with previous studies [23, 35, 47]. Our findings may be explained partly by the Stability of Limiting Elements Hypothesis, which suggests that nutrients required at a high concentration by plants, and which are most frequently considered to be limiting in environments, should be less sensitive to environmental factors [18]. This may imply that leaf N has greater stoichiometric homeostasis even in an N-poor environment. The low soil N level may be explained by the low vegetation coverage and the slow rate of decomposition in arid and saline regions [48, 49], while the higher leaf N concentrations in drought- and salt- tolerant species may be attribute to the higher accumulation of non-protein nitrogen in halophytes in salt-stressed conditions [50, 51]. These non-protein forms of nitrogen, including amino acids, imino acids, proteins, quarternary ammonium compounds, and polyamines, normally account for about 50% of the plant nitrogen content [50, 52], and they play vital roles in osmoprotection and osmotic adjustment [52]. Thus, the higher leaf N level of halophytes appears to indicate an overall trade-off to facilitate saline tolerance, where inefficient N use is exchanged for efficient water use and tolerance of toxic soil salinity levels [53]. Therefore, a higher leaf N content should be a common strategy in desert halophytes.

The halophyte leaf N:P had a significant positive correlation with N (R^2^ = 0.405, *P* < 0.0001), but not with P, which suggests that N may be more limiting for plant growth compared with P in studied habitat. This is consistent with many previous studies in desert ecosystems [49, 54], but different from some others in grassland and woody species [47, 55, 56], which showed that P plays more important roles in plant N:P patterns. In general, limited nutrient availability, plant metabolic rates, and plant functional groups jointly affect the leaf stoichiometry patterns, and thus the relationships between leaf C:N:P stoichiometry and environment differ among various regions [57]. The much higher soil P content compared with coastal saline soils (mean = 1 mg g^-1^) [58] may be attributable to the distinct climates, i.e., more leaching occurs in coastal soil [48]. Thus, the relatively deficiency in soil N content status and the sufficient level of soil P possibly explain why the soil N content is the key limiting factor for the plant N:P ratio in saline desert ecosystems [48, 49].

The significantly lower SLA we found in desert halophytes compared with salt marsh species (*P* < 0.05, S1 Fig.) [53] may support the following hypothesis. Species with higher SLA levels adapt better to resource-rich environments and they have relatively high growth rates [59], whereas the SLA of desert halophytes tends to be lower, but more dry matter is invested per leaf because of the nutrient-poor status of saline desert areas [15, 16]. The LDMC reflects the investment in the persistent leaf structures of plants [60], and thus plants tend to invest more dry matter per leaf in a nutrient-poor environment [15, 16]. Therefore, it is reasonable that a high LDMC and low SLA should be a common strategy for plants living in the low-nutrient and high-salt conditions found in arid saline environments [53].

### Plant functional groups, C:N:P stoichiometry and leaf traits with increasing salinity

We grouped the halophytes according to their salt resistance into succulent woody plants, woody plants, and herbs [28], and found that the herbs were more frequent in low salinity regions, whereas the succulent woody plants were distributed mainly in moderate and severe salinity regions (S1 Table). As a kind of common species in semiarid and arid regions, we had found the succulent woody plants had the lowest leaf C, SLA but highest leaf N. Also they differ greatly from the other two groups in terms of morphology and physiology [5, 61]. For example, in contrast to herb and woody plants, the thick water storage tissues found in the photosynthetic organs of succulent plants may help them to avoid desiccation in drying soil [17]. Moreover, leaf succulence may also improve the energy returns from leaf investment by replacing expensive C structures with water [62], as well as allowing increased C investment in mechanisms that facilitate saline tolerance [53]. This variation of dominant plant group might reflect the response of the saline ecosystem to environmental change with salinization.

In contrast to the dominant species patterns, there were no significant changes in the leaf morphological traits and C:N:P stoichiometry of desert halophytes with different salinity levels. The distribution of salt in different soil layers might explain these results. In desert saline environments, the salt is mainly concentrated in the upper soil layer due to the high rate of evaporation (S2 Fig.) [7]. Thus, we speculate that deep-rooted plants, i.e., most of the plants we investigated, could survive by extending their roots to a low salinity underground region [63], thereby avoiding severe saline stress in the upper soil layers.

Soil extractable N and P, which are the available nutrients that can be absorbed directly by plants, explained larger contributions to the total variance in the leaf N and P, respectively, than the soil salinity levels. This is also supported by the speculation that plants actively avoid the high salinity levels in the upper soil layer [64]. Plant functional groups explained more of the total variation in leaf C than the total soil C and soil salinity, which is expected possibly because the C in plants is derived mainly from the atmosphere via photosynthesis rather than being absorbed from the soil [5, 34]. Similarly, salinity also contributed little to the variation in SLA and LDMC, whereas species explained most of the variation in leaf morphological traits. This agrees with a previous study of Chinese grassland plants [65], which showed that the patterns of leaf traits are influenced jointly by both taxonomic identity and environmental factors, such as climate and soil, whereas leaf traits varied little within species, despite strong variation in the climate and soil conditions [65]. Our study suggests a relative stability in leaf traits and leaf C:N:P stoichiometry of desert halophytes in various saline habitats, although the dominant plant species changed with soil salinity levels. A larger scale investigation with more wide saline gradient in arid and semi-arid saline environment and artificial controlled experiment might provide more details.

## Conclusions

In this study, we found that the general patterns of leaf traits and leaf C:N:P stoichiometry in arid desert halophytes in saline environments agreed to some extent with the trends identified based on analyses of Chinese and global flora, as well as regional investigations of grassland and mountain vegetation. However, some unique patterns were also identified in desert halophytes, as follows. (1) The desert halophytes were characterized by low leaf C and SLA levels, high N levels, but stable P and N:P. The analysis of the correlations between leaf N and P, and N:P indicated the greater degree of N limitation for arid desert halophytes. (2) The variable relationships between leaf morphological traits and the leaf C, N, and P contents at different levels among all observations and each functional group demonstrated that plant functional groups may play more important roles in nutrient fluxes and the response of vegetation to environmental change. (3) Soil available nutrients, but not salinity, were potential drivers of halophyte leaf C:N:P stoichiometry, whereas species made the highest contributions to leaf morphological variation. Giving the complex relationships between salt and vegetation, large-scale investigations of arid and semiarid saline environments, as well as artificially controlled experiments, should be conducted to elucidate the responses of functional groups to soil salinity and the drivers of vegetation variation in increasingly saline environments.

## Supporting Information

S1 Table Description of the 23 sites where samples were collected to assess the leaf traits of plant and to make C, N, P concentration measurements. (DOC)

S2 Table Species included in this study (18 halophytes in total) and average values (arithmetic) of the C:N:P (mass ratio), mass-based leaf carbon (C_mass_), nitrogen (N_mass_), phosphorus (P_mass_) concentrations, SLA, and LDMC. (DOC)

S3 Table Values of soil traits in the study area. (DOC)

S1 Fig Comparison of leaf SLA between desert halophytes and salt marsh plants. The data for salt marsh plants were derived from Eallonardo (2013). The SLA values were averaged by species. The SLA of salt marsh species were significant higher than the desert halophytes in this study (*P* < 0.05)
(TIF)

S2 Fig Soil EC values at different depths. **The EC values of soil samples decreased with soil depth**. There were greater differences in the salinity level of the top soil (0–20 cm) and the other layers. However, the soil EC was almost the same in deeper layers (60–80 cm). Differences of the soil EC values with increased depth within each saline level were tested using one-way ANOVA, significant differences at *P* < 0.05 are indicated by different letters.
(TIF)
