# Supplementary material for: C:N:P Stoichiometry and Leaf Traits of Halophytes in an Arid Saline Environment, Northwest China
Source: PLoS One. 2015 Mar 23;10(3):e0119935. doi: 10.1371/journal.pone.0119935 (PMC4370893; doi:10.1371/journal.pone.0119935)
Supplement: S1 Table — (DOC) [file pone.0119935.s001.doc]

**Table S1. Description of the 23 sites where samples were collected to assess the leaf traits of plant and to make C, N, P concentration measurements.**

| Salinity  level | Site | Soil EC | Vegetation  coverage | Vegetation type | Dominant species |
| --- | --- | --- | --- | --- | --- |
| L1 | YM3 | 1.16 | 40% | Salt meadow | *Agropyron cristatum (H)* |
| L1 | QS3 | 2.61 | 25% | Desert meadow | *Alhagi sparsifolia (H)* |
| L1 | CM1 | 0.73 | <5% | Desert meadow | *Reaumuria soongarica (WS)* |
| L1 | CM2 | 0.90 | <10% | Desert meadow | *Salsola passerine (WS)* |
| L1 | CM3 | 0.46 | 40% | Desert meadow | *Phragmites australis (H)* |
| L1 | CM4 | 0.12 | 43% | Desert meadow | *Phragmites australis (H)* |
| L1 | ST2 | 2.24 | 80% | Desert meadow | *Sophora alopecuroides (H)* |
| L2 | YM1 | 9.47 | 39% | Salt meadow | *Agropyron cristatum (H)* |
| L2 | QS4 | 7.91 | 54% | Salt shrubland | *Phragmites australis (H)* |
| L2 | BH2 | 9.71 | 44% | Salt shrubland | *Alhagi sparsifolia (H)* |
| L2 | BH3 | 8.35 | 52% | Salt meadow | *Phragmites australis (H)* |
| L2 | ST1 | 5.18 | 49% | Desert meadow | *Agropyron cristatum (H)* |
| L3 | YM2 | 13.12 | 36% | Salt meadow | *Tamarix leptostachya (W)* |
| L3 | QS1 | 15.03 | 33% | Salt meadow | *Alhagi sparsifolia (W)* |
| L3 | QS2 | 16.75 | 51% | Salt meadow | *Tamarix leptostachya (W)* |
| L3 | BH1 | 14.18 | 30% | Salt shrubland | *Tamarix leptostachya (W)* |
| L3 | BH5 | 14.48 | 42% | Salt shrubland | *Tamarix leptostachya (W)* |
| L3 | QZ2 | 16.37 | 47% | Salt meadow | *Lycium ruthenicum (WS)* |
| L4 | BH4 | 30.00 | 55% | Salt shrubland | *Lycium ruthenicum (WS)* |
| L4 | QZ1 | 37.27 | 30% | Salt meadow | *Lycium ruthenicum (WS)* |
| L4 | XH1 | 22.57 | 25% | Salt shrubland | *Halostachys caspica (WS)* |
| L4 | XH2 | 48.88 | 25% | Salt shrubland | *Halostachys caspica (WS)* |
| L4 | XH3 | 35.48 | 30% | Salt shrubland | *Halostachys caspica (WS)* |

Soil EC, soil electrical conductivity (ds/m). H: herb species, W: woody species with non-succulent leaves, WS: woody species with succulent leaves. Among the four salinity levels, herb species were dominant in level 1 and level 2, woody species were dominant in level 3, and succulent were dominant in level 4.
