# Supplementary material for: C:N:P Stoichiometry and Leaf Traits of Halophytes in an Arid Saline Environment, Northwest China
Source: PLoS One. 2015 Mar 23;10(3):e0119935. doi: 10.1371/journal.pone.0119935 (PMC4370893; doi:10.1371/journal.pone.0119935)
Supplement: S2 Table — (DOC) [file pone.0119935.s002.doc]

**Table S2. Species included in this study (18 halophytes in total) and average values (arithmetic) of the C:N:P (mass ratio), mass-based leaf carbon (C_mass_), nitrogen (N_mass_), phosphorus (P_mass_) concentrations, SLA, and LDMC。**

| Species | Family | FG^1^ | SLA | LDMC | C_mass_ | N_mass_ | P_mass_ | C:N:P |
| --- | --- | --- | --- | --- | --- | --- | --- | --- |
|  |  |  | (m^2^ kg^-1^) | (g g^-1^) | (mg g^-1^) | (mg g^-1^) | (mg g^-1^) | (mass) |
| *Achnatherum splendens* | Poaceae | H | 7.40 | 0.36 | 421.99 | 19.70 | 1.64 | 257:12:1 |
| *Agropyron cristatum* | Poaceae | H | 10.47 | 0.32 | 421.41 | 24.71 | 1.78 | 237:14:1 |
| *Alhagi sparsifolia* | Fabaceae | H | 9.59 | 0.23 | 420.56 | 33.69 | 1.92 | 219:18:1 |
| *Apocynum venetum* | Fabaceae | WS | 10.60 | 0.23 | 420.94 | 20.78 | 1.60 | 263:13:1 |
| *Asterothamnus centraliasiaticus* | Asteraceae | W | 8.89 | 0.26 | 434.43 | 29.07 | 2.60 | 167:11:1 |
| *Glycyrrhiza inflata* | Apocynaceae | WS | 13.77 | 0.28 | 430.27 | 35.40 | 1.93 | 223:18:1 |
| *Halostachys caspica* | Chenopodiaceae | WS | 5.23 | 0.24 | 337.64 | 42.19 | 2.24 | 151:19:1 |
| *Inula caspica* | Asteraceae | H | 11.33 | 0.20 | 413.09 | 20.50 | 1.83 | 226:11:1 |
| *Lycium ruthenicum* | Solanaceae | WS | 7.73 | 0.15 | 348.92 | 28.75 | 1.82 | 192:16:1 |
| *Nitraria sibirica* | Zygophyllaceae | WS | 9.97 | 0.14 | 377.49 | 47.01 | 2.60 | 145:18:1 |
| *Phragmites australis* | Poaceae | H | 10.55 | 0.36 | 405.02 | 26.22 | 1.64 | 247:16:1 |
| *Populus euphratica* | Salicaceae | W | 7.58 | 0.34 | 444.96 | 18.58 | 1.61 | 276:12:1 |
| *Reaumuria soongarica* | Tamaricaceae | WS | 4.59 | 0.25 | 332.21 | 24.63 | 1.59 | 209:15:1 |
| *Salsola arbuscula* | Chenopodiaceae | WS | 8.26 | 0.17 | 378.97 | 44.26 | 2.79 | 136:16:1 |
| *Salsola passerine* | Chenopodiaceae | WS | 3.56 | 0.18 | 286.80 | 21.27 | 1.67 | 172:13:1 |
| *Sophora alopecuroides* | Fabaceae | H | 11.57 | 0.29 | 457.51 | 40.81 | 1.88 | 243:22:1 |
| *Tamarix leptostachya* | Tamaricaceae | W | 6.12 | 0.37 | 389.39 | 24.66 | 1.76 | 221:14:1 |
| *Zygophyllum xanthoxylon* | Zygophyllaceae | WS | 5.92 | 0.12 | 340.83 | 22.66 | 1.82 | 187:12:1 |

^1^FG, functional group: H: herb species, W: woody species with non-succulent leaves, WS: woody species with succulent leaves. All species in this study were perennial and deciduous.
