# Supplementary material for: C:N:P Stoichiometry and Leaf Traits of Halophytes in an Arid Saline Environment, Northwest China
Source: PLoS One. 2015 Mar 23;10(3):e0119935. doi: 10.1371/journal.pone.0119935 (PMC4370893; doi:10.1371/journal.pone.0119935)
Supplement: S3 Table — (DOC) [file pone.0119935.s003.doc]

**Table S3. Values of soil traits in the study area.**

| Soil traits | Range | Min | Max | Mean | SD | *CV* |
| --- | --- | --- | --- | --- | --- | --- |
| pH | 1.16 | 7.09 | 8.25 | 7.83 | 0.29 | 0.04 |
| *θ*_g_ | 0.25 | 0.02 | 0.27 | 0.16 | 0.08 | 0.50 |
| EC_1:5_ | 48.76 | 0.12 | 48.88 | 12.24 | 12.51 | 1.02 |
| STC | 26.98 | 20.51 | 47.49 | 34.46 | 7.11 | 0.21 |
| STN | 1.06 | 0.14 | 1.20 | 0.54 | 0.34 | 0.63 |
| STP | 5.67 | 2.19 | 7.86 | 5.00 | 1.36 | 0.27 |
| SEN | 5.91 | 0.07 | 5.98 | 0.84 | 1.14 | 1.35 |
| SEP | 28.98 | 0.35 | 29.33 | 7.28 | 7.26 | 1.00 |

The mean values were calculated as the arithmetic means. *θ*_g_, soil water content (g g^-1^); EC1:5, soil electrical conductivity (ds/m); STC, STN, and STP represent the total soil C, N, and P concentrations, respectively (mg g^-1^); SEN and SEP represent the soil extractable N and P, respectively (mg kg^-1^); *CV*, coefficient of variation (SD/mean).
