# Supplementary figures and images for: C:N:P Stoichiometry and Leaf Traits of Halophytes in an Arid Saline Environment, Northwest China
Source: PLoS One. 2015 Mar 23;10(3):e0119935. doi: 10.1371/journal.pone.0119935 (PMC4370893; doi:10.1371/journal.pone.0119935)

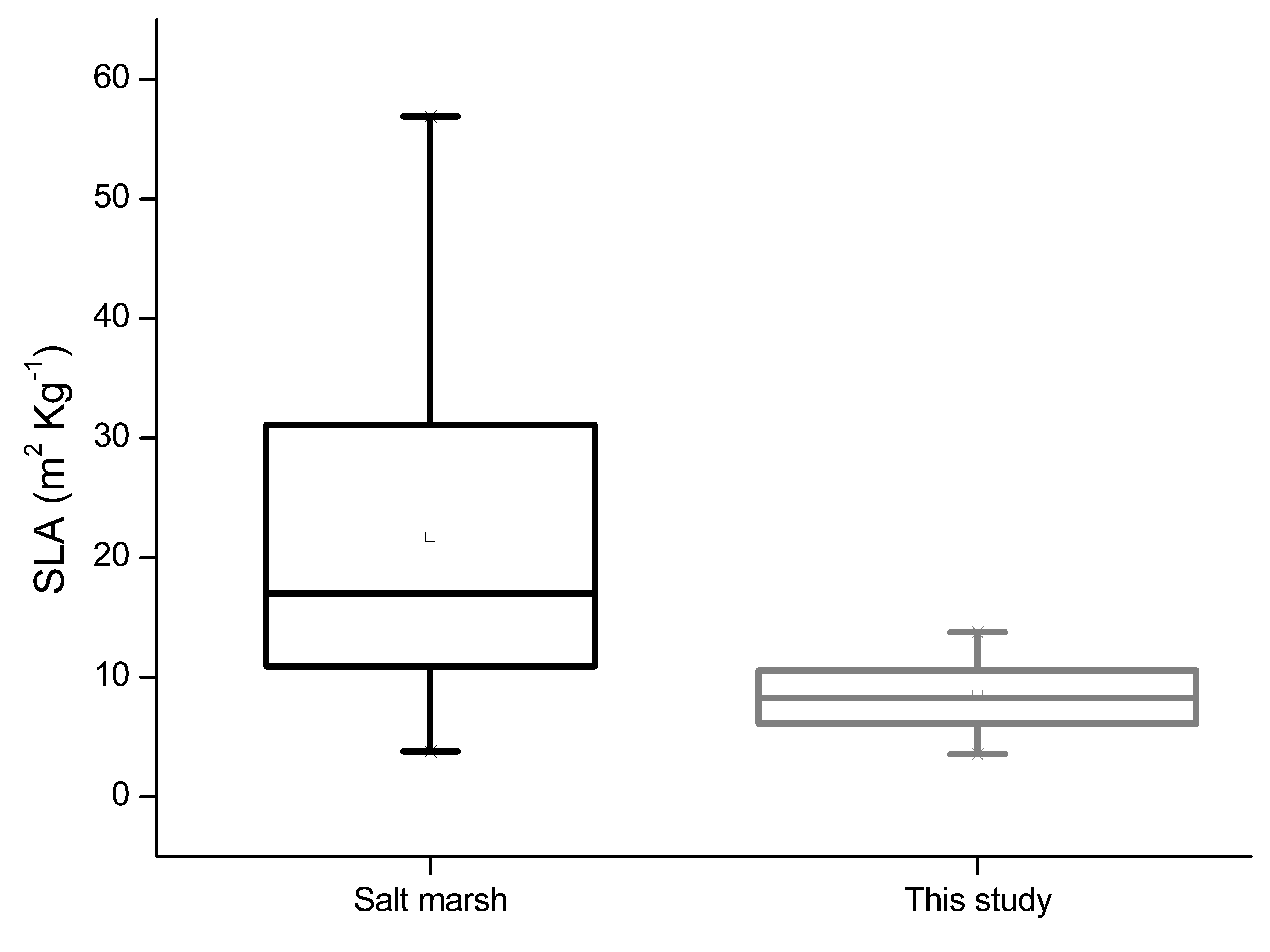

Supplement: S1 Fig — The data for salt marsh plants were derived from Eallonardo (2013). The SLA values were averaged by species. The SLA of salt marsh species were significant higher than the desert halophytes in this study (P < 0.05) (TIF) [file pone.0119935.s004.tif]

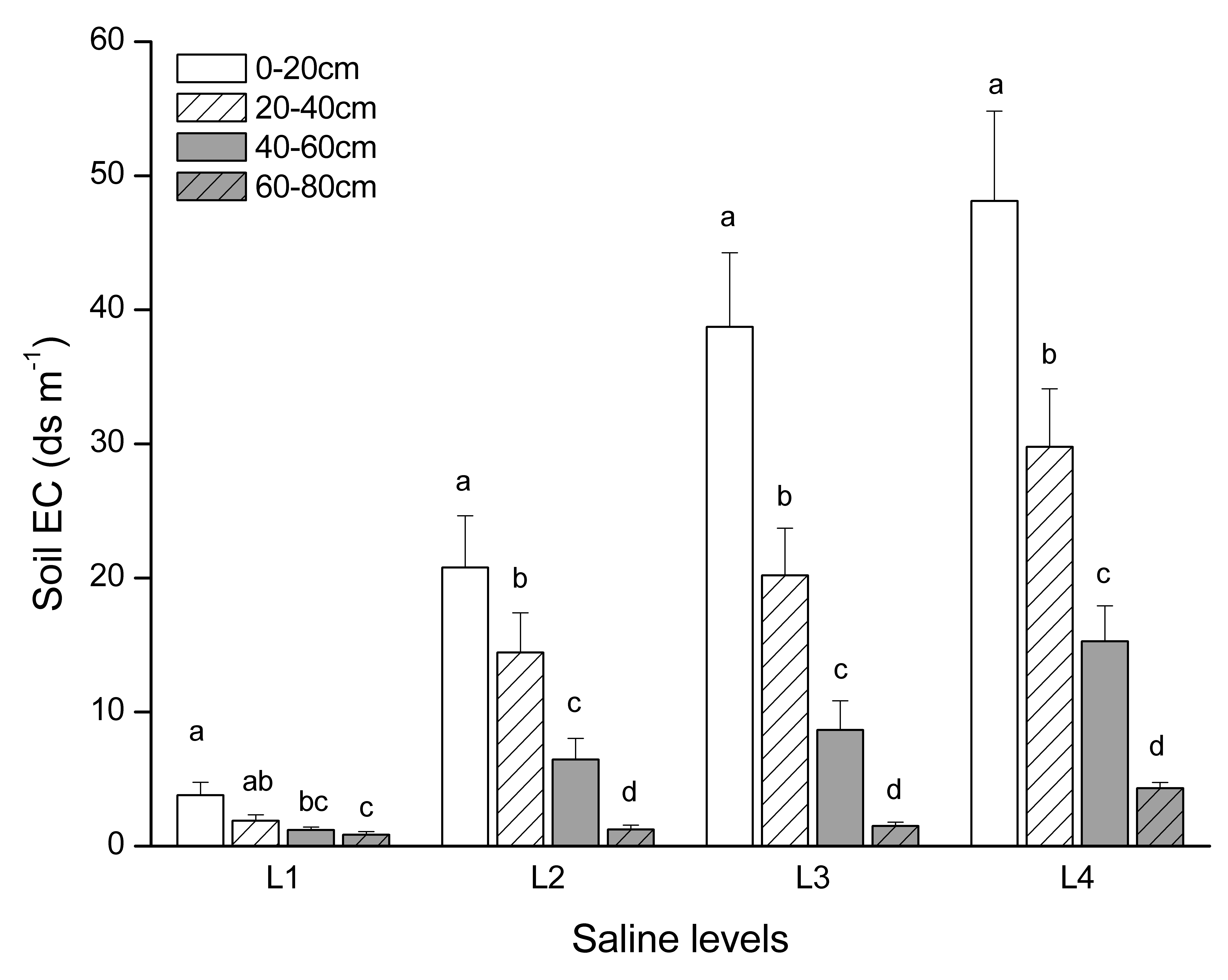

Supplement: S2 Fig — The EC values of soil samples decreased with soil depth. There were greater differences in the salinity level of the top soil (0–20 cm) and the other layers. However, the soil EC was almost the same in deeper layers (60–80 cm). Differences of the soil EC values with increased depth within each saline level were tested using one-way ANOVA, significant differences at P < 0.05 are indicated by different letters. (TIF) [file pone.0119935.s005.tif]
